# NADH Dehydrogenases in *Pseudomonas aeruginosa* Growth and Virulence

**DOI:** 10.3389/fmicb.2019.00075

**Published:** 2019-02-05

**Authors:** Angela Torres, Naomi Kasturiarachi, Matthew DuPont, Vaughn S. Cooper, Jennifer Bomberger, Anna Zemke

**Affiliations:** ^1^Division of Pulmonary, Allergy and Critical Care Medicine, Department of Medicine, University of Pittsburgh, Pittsburgh, PA, United States; ^2^Department of Microbiology and Molecular Genetics, University of Pittsburgh, Pittsburgh, PA, United States

**Keywords:** *Pseudomonas aeruginosa*, NADH dehydrogenase, NADH:ubiquinone oxidoreductases, metabolism, aminoglycoside resistance

## Abstract

*Pseudomonas aeruginosa* is an opportunistic human pathogen with a complex respiratory chain. The bacterium is predicted to express three NADH:ubiquinone oxidoreductases (NDH-1, NDH-2 and Nqr). We created deletions strains of the predicted NADH:ubiquinone oxidoreductases alone, and in combination to determine the respective roles of the NADH dehydrogenases in growth and virulence. NDH-1 and NDH-2 were largely redundant under aerobic conditions. Aerobic NADH dehydrogenase enzymatic activity assay was lost with deletion of both NDH-1 and NDH-2. Under anaerobic conditions, NDH-1 was required for robust growth, and overexpression of NDH-2 rescued the NDH-1 anaerobic growth defect in rich media. There was not compensatory upregulation of NDH-2 under anaerobic conditions in NDH-1 deletion strains. To test which genes were required for *in vivo* virulence, we used both an insect and plant disease model. In the *Galleria mellonella* model, neither deletion of NDH-1 nor NDH-2 led to a change in median lethal dose, although death occurred more slowly in the NDH-1 deletion infections. In a lettuce model of virulence, loss of NDH-1 caused a decrease in recovered viable bacteria and a decrease in visual tissue damage. The compound deletion of NDH-1/NDH-2 causes a severe growth defect, both under aerobic and anaerobic conditions, and was avirulent in a lettuce model. Together, these results demonstrate the redundancy of the *P. aeruginosa* respiratory chain at the NADH dehydrogenase level in aerobic growth and virulence.

## Introduction

*Pseudomonas aeruginosa* is an opportunistic pathogen that causes pneumonia, chronic airway infections, and urinary tract infections (Magill et al., 2014; Crull et al., 2018). The bacterium is also ubiquitous in both soil and water environments. To subsist in this wide range of environments, *P. aeruginosa* is able to metabolize a wide range of carbon sources. The organism has a highly branched respiratory chain with multiple terminal oxidases and can respire both with oxygen and nitrogen oxides (Arai, 2011; Arai et al., 2014). Additionally, there are up to 17 predicted dehydrogenases that may be coupled to the quinone pool, though the physiological role of these enzymes remains poorly understood (Williams et al., 2007). The *P. aeruginosa* genome encodes at least three bioinformatically predicted NADH dehydrogenases (NADH:quinone oxidoreductases). NDH-1, encoded by the *nuoA-N* operon (PA2637-2649), is homologous to the mitochondrial complex I, and has a fused *nuoCD* subunit (Spero et al., 2016). NDH-1 enzymes both translocate protons and oxidize NADH to NAD^+^ (Williams et al., 2007). Previously, it had been reported that the *nuo* operon was required for anaerobic growth through two transposon screens (Filiatrault et al., 2006; Schurek et al., 2008). Mutations within the *nuo* operon are also found in association with aminoglycoside resistance, presumably due to the energy dependency of aminoglycoside uptake (Amini et al., 2011; Ezraty et al., 2013; Yen and Papin, 2017).

In addition to NDH-1, *P. aeruginosa* is also predicted to express one type-II NADH dehydrogenase (NDH-2), which is encoded by the *ndh* gene (PA4538). Type-II bacterial NADH dehydrogenases use FAD as a cofactor to oxidize NADH without translocating protons. NDH-2 enzymes have been a recent focus for antimicrobial development because many microbial organisms lack NDH-1 (Cook et al., 2014; Heikal et al., 2014). The roles of NDH-2 in *P. aeruginosa* growth and virulence are not known. Finally, the *nqrA-F* operon (PA2994-2999) has been experimentally demonstrated to be a unique sodium regulated, proton pumping NADH dehydrogenase in *P. aeruginosa* (Raba et al., 2018). Again, the physiologic role of NQR in *P. aeruginosa* is unclear. During chronic infection, anaerobic growth conditions are common, and core bacterial metabolism during infection is incompletely understood (Alvarez-Ortega and Harwood, 2007). Thus, the purpose of this paper was to determine the respective roles of the NADH: quinone oxidoreductase enzymes in *P. aeruginosa* physiology and *in vivo* virulence.

## Materials and Methods

### Reagents

Lysogeny broth (LB) with 5% NaCl, LB agar, KCN, sodium succinate, sodium acetate, sodium citrate, L-proline, L-glutamine, glucose and potassium nitrate were purchased from Sigma. Other reagents include Muller Hinton Broth Agar (Fluka), M9 powdered salts (BD), gentamicin (Atlanta Biologicals), Breath-Easy Membranes (Diversified BD Biotech), XTT (2,3-Bis-2-Methoxy-4-Nitro-5-Sulfophenl-2-Tetrazolium-5-Carboxanilide from Caymen chemicals) and nicotinamide adenine dinucleotide (NADH from Caymen chemicals).

### Strain Construction

Deletion mutants were made using two-step allelic exchange as described by Hmelo in the PAO1 and PA14 backgrounds (Hmelo et al., 2015). Strains, primers, and plasmids are found in Supplementary Table 1. The following protocol modifications were made: the plasmid pMQ30 was used for allelic replacement (Shanks et al., 2006). 500–1000 bp upstream and downstream of the region to be deleted were amplified by PCR, joined through sequential overlap extension PCR for the allelic replacement construct (ARC) for the NADH dehydrogenase deletion mutants. The Δ*roxSR* ARC was made using NEB Assembly per the kit instructions (New England Biolabs). Ligation mixtures were transformed into *Escherichia coli* DH5α, colonies were screened for the inserts by PCR, and plasmid was isolated from colonies by Miniprep (Qiagen). Inserts were sequence confirmed (Eurofins) and mated into the *P. aeruginosa* recipient strain. Complementation was done by inserting the *ndh* or *nuoIJ* sequence of interest into the pUC18-mini-Tn7 plasmid containing the constitutive P*_nptII_* promoter and sequence confirmed prior to insertion into *P. aeruginosa* (Choi and Schweizer, 2006; Melvin et al., 2017). Transposon insertion mutants were from the PA14 library^1^. Strains were grown overnight in 5 ml of LB on a roller drum prior to experiments except for the Δ*nuoIJ*Δ*ndh* and Δ*nuoIJ*Δ*ndh*Δ*nqr* strains which required 48 h to achieve sufficient culture density for experimentation.

### Sequencing Analysis

The initial deletion was identified through whole genome sequencing on a NextSeq 500 System (Illumina) using 2 × 150 bp libraries. Breseq version 0.28.1 was used for variant calling with alignment to the PAO1 genome (Deatherage and Barrick, 2014). After identification of the initial suppressor, all subsequent deletion strains were subjected to whole genome sequencing.

### Microarray Analysis

Published microarrays were analyzed using the GEO2R tool on the Gene Expression Omnibus (GEO) website (Edgar, 2002). Default settings were used, including the use of the Benjamini and Hochberg false discovery rate method and log transformation of data.

### Gentamicin Minimum Inhibitory Concentration (MIC)

Agar dilution MIC assays using Muller-Hinton Broth agar were performed as described (Wiegand et al., 2008), with the modification that gentamicin concentrations were varied by 0.1 to 0.3 mg/L for quantitative MICs and by 50–100 mg/L for strains containing *aacC1* cassettes.

### Aerobic Growth Curves

Strains were diluted in fresh LB, M9 with glucose or M9 with succinate and inoculated in 3–6 replicates in a 96 well plate. The plates were incubated at 37°C with a gas permeable membrane covering the top of the plate. The plate was shaken for 3 s prior to each OD_600_ measurement on a SpectraMax M2 plate reader (Molecular Devices). Experiments were done 3–6 times.

### Anaerobic Growth Assays

Overnight cultures were grown in LB, mixed well, diluted 1:500 and spotted in 5 μl drops on plates. For anaerobic growth experiments, the following solid media was used supplemented with 1% KNO_3_: LB agar, M9 with sodium succinate, glucose, sodium citrate, ethanol, L-proline, L-glutamine, sodium acetate. Plates were grown in GasPak (BD Biosystems) jars at 37°C for 1–7 days as indicated. Oxygen detection strips were included to insure anaerobic conditions, and in some experiments Δ*anr* strains were included as negative growth controls. For anaerobic growth curves, strains were diluted to an optical density of 0.0005 in media and sealed in Wheaton serum vials. At indicated intervals, vials were sampled aseptically with a needle and syringe and the optical density was measured on a plate reader at 600 nm (OD_600_). Lysogeny broth, M9 with glucose and M9 with succinate were used in these experiments, all supplemented with 1% KNO_3_ to support anaerobic respiration.

### Transcriptional Reporter Assays

We followed the protocol described by Melvin et al. (2017) with the following modifications. The 300 base pair intergenic region upstream of *ndh* was inserted into the transcriptional fusion reporter plasmid pAG4 using NEBAssembly (New England Biolabs). The sequenced plasmid was inserted into a single site on the *P. aeruginosa* chromosome (Glassing and Lewis, 2015). Plasmid with no insert was used as a negative control, and the P*_ntpII_* promoter was used as a positive control. For aerobic assays, overnight cultures of reporter strains were diluted in fresh Lysogeny Broth, grown to mid-log phase and luminescence was measured. For anaerobic studies, overnight cultures were grown on a roller drum and then diluted 1:50 in LB with 1%KNO3. The Δ*nuoIJ* and Δ*anr* strains were diluted 1:10 so that similar culture density would result at the end of the assay. Subcultures were incubated in a GasPak jar for 6 h, at least 2 h after the indicator strips indicated oxygen depletion. Luminescence was measured using a Synergy 2 plate reader (BioTek), and measurements were normalized to OD_600_ and empty vector signal intensity for each day. The experiment was done in triplicate.

### Enzymatic Activity Assays

Fifty milliliter overnight cultures were grown in LB with shaking. Bacteria were pelleted and sonicated in 0.1 M Tris buffer pH 7.5. Cell debris was removed via low speed centrifugation, and the supernatant was centrifuged at 32,000 × g for 10 min to pellet the membrane fraction. NADH was measured spectrophotometrically at 340 nm. Results were normalized to protein concentration. The assay was performed 10 times, and results are shown normalized to the parental strain activity for each replicate with 95% confidence intervals. The NADH-Ubiquinone oxidoreductase assay was used with 1 μM antimycin A, 5 mM KCN, and XTT (a formazan dye) as a terminal electron acceptor. Membranes were incubated with these reagents in 0.1 M Tris buffer, pH 7.5 at 37°C and formation of formazan dye product was monitored spectrophotometrically over 30 min. Equal protein was loaded for both assays. Results shown as means with 95% confidence intervals.

### Galleria Virulence Model

The protocol described by Jander was used with stationary phase cultures (Jander et al., 2000). *G. mellonella* larvae were obtained from Grubco (Fairfield, OH) and kept at room temperature. Overnight bacterial cultures were rinsed in 10 mM MgSO_4_, diluted, and injected into the larvae using a 30-gauge needle. For survival curves, live worms were counted every 2 h during the period frequent deaths were occurring, and then every 4–12 h. For LD_50_ determinations, worms were injected with varying inocula and live worms were counted at 72 h. Sterility controls were included in all experiments. Median lethal infections inocula were determined through probit analysis.

### Lettuce Virulence Model

The protocol described by Starkey was used (Starkey and Rahme, 2009). Romaine lettuce was purchased the morning of the experiment. Overnight cultures were rinsed in 10 mM MgSO_4,_ diluted to an optical density at 600 nm (OD_600_) of 0.2 and injected into the rib of the lettuce leaf. Leaves were incubated at room temperature and assessed daily for visual signs of infection. After 4 days, the infected portion of leaf was excised, homogenized with a bead beater, and colony forming units (CFU) were plated by serial dilution. Experiments were performed 3–4 times with at least 3 replicate leaves per strain in each experiment.

### Statistics

Prism version 7.0 software (GraphPad Software, Inc.) was used to display and analyze data. Number of replicates for experiments is described in figure legends, and at least three replicates were done for each experiment. Tests are indicated in figure legends. When applicable, one-way ANOVA tests were used with *post hoc* Dunnett test to compare multiple groups. For assays that were normalized to control values, a one-way *t*-test was done comparing to the theoretical mean of 1.0. Survival analysis was used for the *G. mellonella* experiment, with the log-rank test used to compare survival curves. Probit analysis was used to determine LD_50_.

## Results

### Role of NDH-1 in Anaerobic Growth and Aminoglycoside Resistance

**Figure 1:**
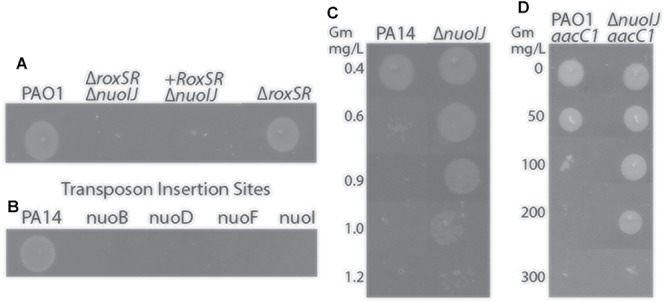
**(A)** Deletion of *roxSR* caused an anaerobic growth defect on LB with 1% KNO_3_ that failed to complement. When sequencing revealed deletion of *nuoIJ*, the reconstructed Δ*roxSR* strain grows anaerobically. **(B)** Transposon mutants within the *nuo* operon fail to grow anaerobically. **(C)** The gentamicin MIC for PA14 is 0.6 mg/L, while the MIC for PA14 Δ*nuoIJ* is increased to 1.2 mg/L. **(D)** The *aac1* cassette inserted to a single copy chromosomal site in combination with *nuoIJ* deletion increases the MIC from 100 to 300 mg/L.

We initially encountered a large deletion in the *nuo* operon (NDH-1) while constructing a deletion mutant of *roxSR* (PA4493-4494) using two-step allelic exchange with gentamicin as the selection agent for an unrelated study (Hmelo et al., 2015). The two-component sensor kinase RoxSR regulates aerobic terminal oxidase expression, however it was not reported to regulate pathways required for anaerobic growth (Kawakami et al., 2010). The resulting Δ*roxSR* strain exhibited an anaerobic growth defect that could not be complemented despite multiple approaches (Figure 1A). Because complementation failed, we subjected the strain to whole genome sequencing and found a 759 bp deletion spanning *nuoIJ*, in addition to the expected deletion of *roxSR.* The strain was reconstructed, and the reconstructed Δ*roxSR* strain did not have an anaerobic growth defect, which suggested that the *nuoIJ* deletion caused the anaerobic growth defect. It has been previously reported that transposon insertion within the *nuo* operon gives an anaerobic growth defect, and we were able to replicate these results using strains from the PA14 ordered transposon mutant library (Figure 1B; Filiatrault et al., 2006). NDH-1 in prokaryotes consists of a two protein arms: membrane and peripheral. The *nuoIJ* deletion spans both arms and is thus predicted to disrupt protein function. We used a Δ*nuoIJ* strain throughout due to the difficulty in complementing such a large operon in its entirety. The *nuoIJ* deletion was encountered while using gentamicin as a selection agent, and because the *nuo* operon has previously been reported to be under genetic pressure after exposure to aminoglycosides, we tested if deletion of *nuoIJ* resulted in an increase in gentamicin MIC using agar dilution (Yen and Papin, 2017). The MIC of PA14 was 0.4 mg/L and the MIC of PA14 Δ*nuoIJ* increased 3-fold to 1.2 mg/L (Figure 1C). The complemented strain was not tested because it contained a gentamicin resistance cassette. Similar results were obtained in the PAO1 background. Notably, the gentamicin MICs for the Δ*ndh*, Δ*nqrA-F*, and Δ*ndh* Δ*nqrA-F* strains were within 0.3 mg/L of the parental strain, and the Δ*nuoIJ*Δ*nqrA-F* strain MIC was within 0.3 mg/L of the Δ*nuoIJ* strain (data not shown). The slow growth of the Δ*nuoIJ*Δ*ndh* and Δ*nuoIJ*Δ*ndh*Δ*nqrA-F* strains complicated interpretation of the MIC assays, however the MIC for the strains was at least as high as the Δ*nuoIJ* strain. The increase in gentamicin resistance imparted by Δ*nuoIJ* was additive to the *aacC1* cassette that is frequently used for genetic manipulations in *P. aeruginosa*, increasing the MIC of PAO1-*aacC1* from 100 to 300 mg/L (Figure 1D). In summary, we encountered an incidental 759 bp deletion in the *nuo* operon that caused an anaerobic growth defect and increased resistance to gentamicin. Because the genome of *P. aeruginosa* contains multiple putative NADH dehydrogenases, we then determined the role of each in growth and virulence.

### Role of NADH Dehydrogenase Genes in Growth

**Figure 2:**
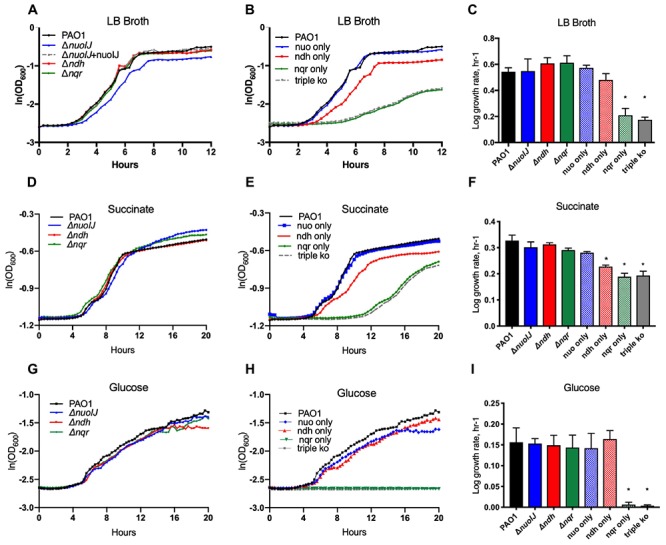
Indicated strains were grown in a microtiter dish on LB, M9 with glucose or succinate as carbon sources. **(A)** In LB, deletion of any single NADH dehydrogenase does not change log phase growth rate. **(B)** The compound Δ*nuoIJ Δndh* (“nqr only”) and Δ*nuoIJ Δndh Δnqr* (“triple ko”) strains grow slowly in LB. **(C)** Maximal log phase growth rate in LB (mean ± SD). **(D)** With succinate as a carbon source, deletion of any individual NADH dehydrogenase did not affect aerobic growth. **(E)** With succinate as a carbon source, the compound Δ*nuoIJ Δndh* (“nqr only”) and Δ*nuoIJ Δndh Δnqr* (“triple ko”) strains grew slowly, and there was a slight decrease in growth rate in a strain expressing only *ndh* (Δ*nuoIJ Δnqr)*. **(F)** Maximal log phase growth rate in succinate (mean ± SD). **(G)** With glucose as a carbon source, deletion of any individual NADH dehydrogenase did not affect aerobic growth. **(H)** With glucose as a carbon source, the compound Δ*nuoIJ Δndh* (“nqr only”) and Δ*nuoIJ* Δ*ndh* Δ*nqr* (“triple ko”) strains showed now growth within 20 h. **(I)** Maximal log phase growth rate in glucose. ^∗^ indicates *p* < 0.01 by one-way ANOVA followed by Dunnett’s multiple comparison test. At least 3 replicates were done for all experiments and representative growth curves are shown.

We determined the role of the NADH dehydrogenase enzymes in aerobic growth. Single deletions of *nuoIJ, ndh*, and *nqrA-F* did not affect log phase growth rate in rich media, minimal media (M9) succinate, or M9 with glucose as carbon sources (Figure 2A,C,D,F,G,I). However, the Δ*nuoIJ* strain grew to slightly lower stationary phase density in LB (PAO1 parental 8.3 ± 6.7 × 10^9^CFU/ml vs. Δ*nuoIJ* 2.5 ± 2.7 × 10^9^ CFU/ml, *p* < 0.05 by two-tailed *t*-test). Strains expressing nqr only (Δ*nuoIJ*Δ*ndh*) or no known NADH dehydrogenases (Δ*nuoIJ*Δ*ndh*Δ*nqrA-F*, triple ko) had a growth defect that varied in severity by carbon source. In rich media and M9-succinate, the Δ*nuoIJ*Δ*ndh* strain (nqr only) had a 30–50% decrease in log phase growth rate compared to the parental strain, and deletion of all three dehydrogenases Δ*nuoIJ*Δ*ndh*Δ*nqrA-F* (triple ko) did not result in a further decrease in growth (Figure 2B,E). However, with glucose as a carbon source, either NDH-1 or NDH-2 was required for growth, with no growth seen in strains expressing only NQR (Figure 2H).

**Figure 3:**
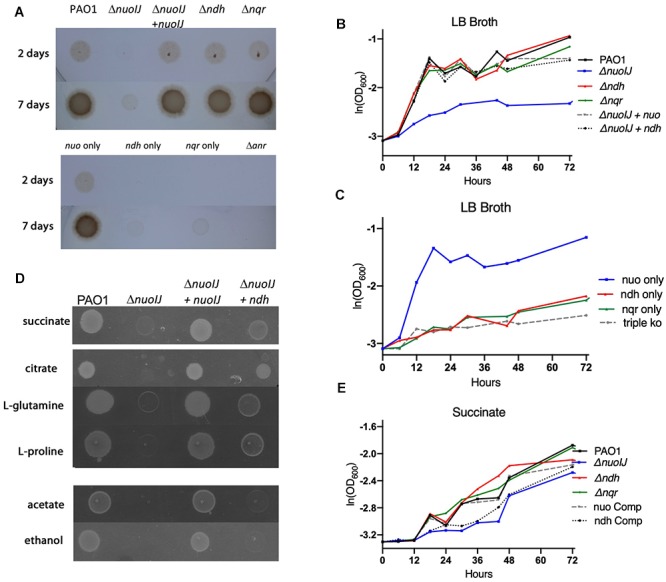
**(A)** Overnight cultures of NADH dehydrogenase mutant strains were diluted 1:500 and grown for one to seven days anaerobically on plates containing 1% KNO_3_. The Δ*nuoIJ* had scant growth at 7 days, that was complemented by *nuoIJ*. **(B)** Strains were diluted in LB broth with 1% KNO_3_, sealed in glass vials and incubated at 37°C. OD_600_ was measured at indicated times. In LB, the Δ*nuoIJ* strain shows little growth. The growth defect was complemented by expression of *nuoIJ* or overexpression of *ndh.* **(C)** In LB, the strain expressing *nuo* only (Δ*nqr* Δ*ndh)* grew anaerobically, while compound deletion strains with *nuoIJ* deleted grew poorly. **(D)** M-9 agar plates with the indicated carbon sources were grown anaerobically for 48 h. The Δ*nuoIJ* had clearly visible growth at 48 h on succinate, faint growth on citrate, L-glutamine and L-proline and no visible growth on acetate and ethanol. Expression of *nuoIJ* complemented growth defects on all carbon sources. Overexpression of *ndh* complemented growth on citrate, L-glutamine and L-proline, but not acetate or ethanol. **(E)** Strains were diluted in M9-succinate containing 1% KNO_3_, sealed in glass vials and incubated at 37°C. OD_600_ was measured at indicated times. The Δ*nuoIJ* strain shows a mild growth defect that is not complemented by overexpression of *ndh.*

We then studied anaerobic growth both qualitatively on solid media (Figure 3A) or in broth quantified by optical density of the culture (Figure 3B,C). Under anaerobic conditions on LB agar supplemented with 1% KNO_3_, the Δ*nuoIJ* strain grew very slowly with scant growth visible by 7 days (Figure 3A). The growth defect was complemented by expression of either *nuoIJ* or *ndh* under the constitutive promoter P*_nptII_* (Figure 3B). Of note, this promoter increases expression of *ndh* approximately 40-fold as compared to baseline (data not shown). Compound mutants containing the Δ*nuoIJ* deletion grew poorly anaerobically, while the strain expression only *nuo* (Δ*ndh*Δ*nqr*) grew similarly to the parental strain (Figure 3C). An Δ*anr* strain was included as a control for anaerobic conditions. ANR is the master transcriptional regulator for denitrification and is required for anaerobic growth (Zimmermann et al., 1991). In minimal media with succinate, deletion of *nuoIJ* resulted in a milder growth defect. Growth was clearly visible on solid media at 48 h (Figure 3D), so growth was quantitated by OD (Figure 3E). Overexpression of *ndh* did not complement the mild growth defect seen in the Δ*nuoIJ* strain (Figure 3E). That NDH-2 complemented NDH-1 on rich media but not on M9 succinate, suggested that the requirement of NADH dehydrogenases for redox balance versus proton motive force (pmf) generation may vary by substrate. Therefore, we tested the growth of these strains on several carbon sources (Figure 3D). Citrate, L-glutamine and L-proline feed into the tricarboxylic acid (TCA) cycle prior to succinate dehydrogenase. On these substrates, an anaerobic growth detect was seen in the Δ*nuoIJ* strain that was complemented by either expression of *nuoIJ* or overexpression of *ndh* (Figure 3D). Ethanol and acetate are not readily converted to succinate. An anaerobic growth defect was seen in the Δ*nuoIJ* strain on both ethanol and acetate, that was not complemented by expression of *ndh* (Figure 3D).

Cumulatively, we saw that loss of *nuoIJ* had minimal effects on aerobic growth in multiple media types, with *ndh* being able to support growth in most situations. Anaerobically loss of *nuoIJ* resulted in a severe growth defect on most carbon sources tested, but a smaller growth defect on succinate. Overexpression of *ndh* was able to complement Δ*nuoIJ* completely in rich media, citrate, L-glutamine and L-proline.

### Analysis of Gene Expression and Enzymatic Activity

**Figure 4:**
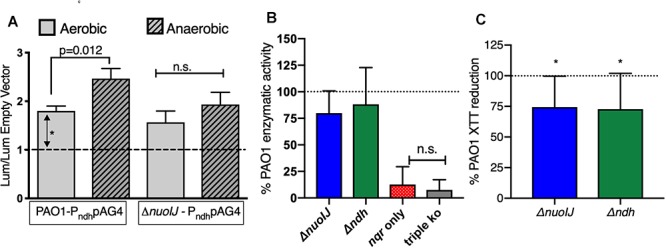
**(A)** Transcriptional reporter strains were made with the 300 bp intergenic region upstream of *ndh* inserted upstream of *lux.* Results shown normalized to empty vector luminescence and optical density of culture. Three replicates shown. *p*-values indicated from one-way ANOVA followed by Dunnett’s test. **(B)** NADH dehydrogenase assay, normalized to parental strain for each day. Ten replicates shown, and *p* < 0.001 for the strain expressing nqr only (Δ*nuoIJ Δndh*) and the triple knockout strain compared to PAO1 by one-way ANOVA. Mean ± 95% CI shown. **(C)** XTT assay in the presence of antimycin and cyanide. Deletion of either Δ*nuoIJ* or Δ*ndh* results in decreased XTT reduction. *p* < 0.05 by one-way *t*-test for each strain. Eight replicates shown. Mean ± 95% CI shown.

One explanation for the growth defect seen with deletion of NDH-1 under anaerobic conditions is that NDH-2 is not expressed anaerobically. The GEO Database contains two microarray data sets comparing gene expression under aerobic and anaerobic conditions (Alvarez-Ortega and Harwood, 2007; Trunk et al., 2010). We examined both data sets using the GEO2R tool, and none of the NADH dehydrogenase operons were regulated in response to oxygen in these data sets. The sensitivity of these data sets using microarray technology may be limited for subtle changes, so we created transcriptional reporter strains fusing the putative *ndh* promoter upstream of luciferase (*lux*) in the plasmid pAG4 (Glassing and Lewis, 2015). Overall aerobic reporter expression was low but reliably detectable at 2-fold as compared to baseline (Figure 4A). In the parental strain there was a modest, but statistically significant, increase under anaerobic conditions. Aerobically, reporter expression was unchanged in the Δ*nuoIJ* as compared to parental, and the increased expression seen anaerobically in the parental strain was blunted (Figure 4A). We then measured NADH dehydrogenase enzymatic activity directly by following loss of NADH spectrophotometrically in the presence of isolated membranes. The Δ*nuoIJ*Δ*ndh* and Δ*nuoIJ*Δ*ndh*Δ*nqrA-F* strains did not have measurable enzymatic activity, while the Δ*nuoIJ* and Δ*ndh* strains did not show a statistically significant decrease in total NADH dehydrogenase activity after ten replicates (Figure 4B). These results strongly indicate that NDH-1 and NDH-2 account for the total NADH dehydrogenase enzymatic activity under the condition tested. Regarding the absence of effect with deletion of NDH-1 or NDH-2 alone, the NADH assay may be limited by downstream capacity of the respiratory chain to accept electrons, thus we were not able to determine if loss of NDH-1 or NDH-2 resulted in decreased total NADH dehydrogenase activity. We then used the chromogenic formazan dye XTT combined with potassium cyanide and antimycin A to block transfer of electrons to the distal respiratory chain to measure NADH dehydrogenase activity. Deletion of either *nuoIJ* or *ndh* resulted in decreased NADH enzymatic activity in this assay (Figure 4C). From these data we concluded that *ndh* is not upregulated under anaerobic conditions in the Δ*nuoIJ* strains, but it does continue to be expressed. NDH-1 and NDH-2 supply the NADH dehydrogenase activity present during growth in rich media.

### *In vivo* Roles of NADH Dehydrogenases in Plant and Insect Infections

**Figure 5:**
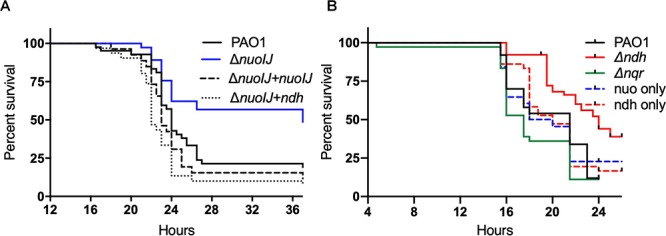
Survival curves for *Galleria mellonella* injected with 10 bacteria/larvae of indicated bacterial strains. **(A)** Δ*nuoIJ* strain has increased survival compared to parental PAO1 or Δ*nuoIJ* complemented with either *nuoIJ* or *ndh* (*p* = 0.002 by Log-Rank test). **(B)** Deletion of *ndh* or *nqr* did not change survival (alone or in combination).

We tested the effects of the deletion of NADH dehydrogenases on virulence in an insect and plant model. In the *Galleria mellonella* waxworm model of insect virulence, the median lethal dose for PAO1, Δ*nuoIJ* and Δ*ndh* were identical (1.3–1.5 infecting bacteria/larva). The compound deletion Δ*nuoIJ*Δ*ndh* caused a small increase in LD_50_ to 8.5 bacteria/larva. While the LD_50_ was unchanged with loss of NDH-1, the kinetics of killing was significantly slower (*p* < 0.001) in the Δ*nuoIJ* strain as compared to PAO1 (Figure 5A and Table 1). Expression of either *nuoIJ* or *ndh* in the Δ*nuoIJ* strain was sufficient to return killing kinetics to that of the parental strain. We did not test killing kinetics in the Δ*nuoIJ*Δ*ndh* strain because the LD_50_ was lower than PAO1. Deletion of *ndh* or *nqr*, alone or in combination, had no effect (Figure 5B).

**Table 1:** Median lethal inocula for NDH deletion strains with 95% confidence intervals.

Strain	LD_50_ (95% CI)
PAO1	1.5 (1.1–2.1)
Δ*nuoIJ*	1.4 (1.0–2.0)
Δ*ndh*	1.3 (0.9–1.8)
Δ*nuoIJΔnndh*	8.5 (4.9–14.7)

**Figure 6:**
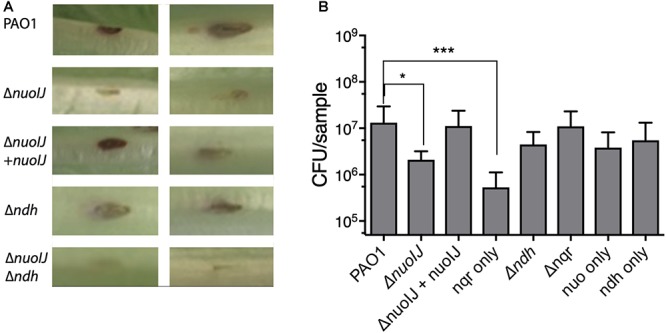
**(A)** Representative images from lettuce virulence model. Infected area appears in brown, and two representative replicates are shown for each strain. **(B)** Quantification of CFU from infected leaf areas. 3–4 independent experiments pooled. Brackets indicate *p* < 0.05 by one-way ANOVA followed by Dunnett’s test.

We then used a lettuce model to test the virulence of the NDH deletion strains (Starkey and Rahme, 2009). Loss of NDH-1 caused a slight decrease in visual tissue damage (Figure 6A). The Δ*nuoIJ* strain had a 1-log decrease in recoverable CFUs at day 4, which was complemented. Deletion of both NDH-1 and NDH-2 caused a larger decrease in virulence (*p* < 0.05). Deletion of *ndh* or *nqr*, alone or in combination, had no effect (Figure 6B). Together, these data suggest that at least one NDH is required for full virulence in the lettuce model, with NDH-1 being the preferred enzyme. Overall, loss of NDH-1 caused a small but measurable decrease in virulence, while deletion of NDH-2 or NQR had no detectable effect.

## Discussion

In this study, we observed that of the NADH dehydrogenases in *P. aeruginosa*, NDH-1 (encoded by the *nuo* operon) is required for anaerobic growth in rich broth and glucose, as well as full virulence in plant and insect pathogenesis models. *In vivo*, loss of NDH-1 led to slower killing of *G. mellonella* larvae and less tissue damage in the lettuce infection model. We did not identify a clear physiologic role for NDH-2 in these studies. The deletion of both NDH-1 and NDH-2 leads to loss of NADH enzymatic activity demonstrating that NDH-2 is biochemically functional and expressed.

We initially encountered a large deletion within *nuo* while using gentamicin as a selection agent during allelic recombination. We saw a 3-fold increase in gentamicin MIC with loss of NDH-1, similar to what has been reported in *E. coli* and *Salmonella.* Aminoglycoside uptake is strongly dependent on proton motive force, to which NDH-1 contributes, thus it is perhaps not surprising that the *nuo* operon was the target for a suppressor while using gentamicin to genetically manipulate *P. aeruginosa.* We did not exclude alternative mechanisms for increased gentamicin resistance such as increased efflux. Mutations in *nuo* are also found during laboratory evolution experiments with aminoglycosides in *P. aeruginosa*, and the operon was identified as a contributor to low-level aminoglycoside resistance through transposon screening (Schurek et al., 2008; Amini et al., 2011; Yen and Papin, 2017). Apart from the change in aminoglycoside resistance, loss of NDH-1 causes a very subtle aerobic phenotype in rich media, and these results demonstrate the utility of affordable, whole genome sequencing as confirmation during the creation of genetically manipulated bacterial strains.

### Redundancy of NDH Enzymes in Aerobic Growth

During aerobic growth any of the individual NADH dehydrogenases are dispensable for log phase growth on glucose, succinate or rich media. As with many studies using deletion strains, the caution that the physiologic role of any enzyme may not be revealed due to compensatory changes due to the deletions themselves applies. Strains possessing only NDH-1 or only NDH-2 grew well in all conditions tested. Strains expressing only NQR grew poorly in all conditions; and we did not detect a difference in growth between the strains expressing only *nqrA-F* and those with all three NADH dehydrogenase operons deleted. Compound deletion of NDH-1 and NDH-2 leads to undetectable NADH dehydrogenase activity under the conditions tested. NQR was recently reported to be a proton pumping NADH dehydrogenase in *P. aeruginosa*, however the physiologic role of the enzyme remains uncertain as compound deletion of NDH-1 and NDH-2 abolished NADH dehydrogenase activity under the conditions studied (Raba et al., 2018). Directly measuring NADH spectrophotometrically, we were unable to detect a change in NADH consumption with deletion of NDH-1 or NDH-2 alone. The NADH assay requires that the rest of the respiratory chain not be limiting, and it is possible that with NDH-2 alone provides enough excess NADH dehydrogenase activity compared to more limiting steps that a reduction was not detected. Using a tetrazolium dye as a terminal electron acceptor with cyanide and antimycin blocking transfer of electrons through cytochrome bc_1_ to the terminal oxidases, we detected a modest decrease in activity in with deletion of either NDH-1 or NDH-2. Despite these limitations, it is clear that NDH-1 and NDH-2 are the main NADH dehydrogenases required for aerobic growth.

### Requirement of NDH-1 for Anaerobic Growth

We observed a profound anaerobic growth defect with deletion of NDH-1 on most substrates. NADH dehydrogenases serve two potential metabolic roles. During canonical anaerobic denitrification only 6 protons are pumped per 2 electrons (Chen and Strous, 2013). NDH-1 pumps 4 of these 6 protons, thus NDH-1 is predicted to significantly contribute to proton motive force generation. Additionally, on some substrates NADH dehydrogenases may be required to redox balance NADH generated by the TCA cycle or other reactions while pmf is at least partially supported through other means. On succinate the growth defect with loss of NDH-1 was milder than on rich media. Complementation studies were difficult to reliably interpret because the role of NDH-1 in growth was less. Compounds that feed into the TCA cycle prior to succinate dehydrogenase (L-proline, L-glutamine and citrate) can contribute to electron transport via complex 2. However, these energy sources also are predicted to generate NADH requiring some means of balancing the redox state of the cell. On these substrates NDH-2, which can provide redox balance but not generate pmf, was able to complement NDH-1. Substrates that cannot be readily converted to succinate (e.g., acetate and ethanol) cannot contribute to pmf via complex 2 and therefore require NDH-1. More than redox balance is required to support growth on these substrates. *P. aeruginosa* displays great metabolic flexibility with a highly branched respiratory chain at the terminal oxidase level. At least five aerobic terminal oxidase are expressed that have a range of oxygen affinities and proton translocation efficiencies. Branching at both the NDH and terminal oxidase levels may increase the flexibility of the chain by allowing the decoupling of pmf generation and regeneration of NAD^+^ (Calhoun et al., 1993; Kerscher et al., 2008; Bekker et al., 2009).

### Redundancy in Virulence

NDH-1 was uniquely required for full *in vivo* virulence. *P. aeruginosa* is highly virulent in waxworms, with an LD_50_ of only a single bacterium. Loss of NDH-1 does not change the LD_50_ but causes a small delay in time to death. We saw a slightly more pronounced phenotype in the lettuce model, with less visible tissue damage and fewer recoverable CFUs with loss of NDH-1. In both the insect and lettuce models, compound deletion of NDH-1 and NDH-2 resulted in a dramatic decrease in virulence. Our results show that NADH dehydrogenase activity is required for virulence, but NDH-1 and NDH-2 may have overlapping functions *in vivo.* Apparent redundancy of function between NDH-1 and NDH-2 in virulence is found in a number of other bacterial species. *Mycobacterium tuberculosis* has one NDH-I and two NDH-2 genes, which were recently shown to be largely functionally redundant, although Δ*ndh* was attenuated in a murine model. *Pseudomonas fluorescens* WCS365 expresses both *nuo* and *ndh* during rhizosphere colonization, and the *nuo* deficient strain was less competitive than the parental strain during colonization assays (Camacho Carvajal et al., 2002). Our findings underscore the need for at least one NDH enzyme for growth and virulence in *P. aeruginosa.* Under aerobic conditions, these enzymes are largely functionally redundant. Anaerobically, NDH-2 is not upregulated to a degree that will support anaerobic growth in the absence of NDH-1. Loss of NDH-1 leads to a slightly decreased virulence. These data reinforce the apparent redundancy and flexibility of the respiratory chain.

## Author Contributions

AT, NK, MD, and AZ acquired the data. AZ, VC, and JB analyzed and interpreted the data. All authors contributed to writing the manuscript.

## Conflict of Interest Statement

The authors declare that the research was conducted in the absence of any commercial or financial relationships that could be construed as a potential conflict of interest.

## Footnotes

1 http://ausubellab.mgh.harvard.edu/cgi-bin/pa14/home.cgi, accessed April 5, 2018.
