# Supplementary material for: NADH Dehydrogenases in Pseudomonas aeruginosa Growth and Virulence
Source: Front Microbiol. 2019 Feb 5;10:75. doi: 10.3389/fmicb.2019.00075 (PMC6370648; doi:10.3389/fmicb.2019.00075)
Supplement: Supplementary file 1 [file Table_1.docx]

**Characterization of NADH Dehydrogenases in Pseudomonas aeruginosa**

Angela Torres, Naomi Kasturiarachi, Matthew DuPont, Vaughn S Cooper, Jennifer Bomberger and Anna Zemke*

Original Research, Front. Microbiol. - Microbial Physiology and Metabolism

Submitted on: 25 Sep 2018

Manuscript ID: 426878

Supplemental Table 1: Strains, Primers and Plasmids Used

| Deletion Primers |  |
| --- | --- |
| RoxUpF | gtaaaacgacggccagtgccaatcggcgatgctgaagtccat |
| RoxUpR | ccccgttgtcatttacgctgttttcttccggaataagcgc |
| RoxDownF | cgccttattccggaagaaaacagcgtaaatgacaacgggggc |
| RoxDownR | gagtcgacctgcaggcatgcaccgtcggtatagagggaga |
| ndhUpF | gcatgagctcCGAGATGCGCCGCAACAT |
| ndhUpR | gtaatggctcagtgcagcttgaggcgatgggacatgtggatatctc |
| ndhDownF | gatatccacatgtcccatcgcctcaagctgcactgagccattac |
| ndhDownR | gcatgagctcgctggctgatggatggctt |
| nqrUpF | ccatgaattccgttctcttcgaaatagttgcg |
| nqrUpR | tcaagctgtcgctggcggagcgtttccgtccgcttg |
| nqrDownF | caagcggacggaaacgctccgccagcgacagcttga |
| nqrDownR | cgtagaattcctggagctggtgggtatcac |
| NuoIJ UpF | cagtGAATTCcagaccatctcctacgag |
| NuoIJ UpR | gtttggcgtcttgacgaccacgacgttgatgatttctttg |
| NuoIJ DownF | caaagaaatcatcaacgtcgtggtcgtcaagacgccaaac |
| NuoIJ DownR | gtctGAATTCgaagttggtgctgaagtcg |
|  |  |
|  |  |
| Complementation Primers | |
| RoxSR insertF | gggGGATCCatcggcgatgctgaagtccat |
| RoxSR InsertR | GGGgaattcTATCCCCGGCGACCGGCGGAA |
| NuoIJ CompF | cagtGGATCCcccagtaaggagaaccacc |
| NuoIJ CompR | cagaAAGCTTcagcgttacgttcccttgg |
| NdhCompF | gctaACTAGTatgtcccatcgcatcgtga |
| NdhCompR2 | gcctAAGCTTtcagtgcagcttgaggcg |
|  |  |
|  |  |
| Plasmids |  |
| pMQ30 | Described in (15) |
| pUC18-mini-Tn7-Gm | Described in (16) |

| Deletion Strains |  |
| --- | --- |
| ΔroxSR | strains made with and without nuoIJ suppressor |
| Δ*nuoIJ* | made in both PAO1 and PA14 |
| Δndh |  |
| Δnqr |  |
| ΔnuoIJΔndh | NuoUPloa |
| ΔnuoIJΔnqr | expresses ndh only |
| ΔndhΔ nqr | expresses nuo only |
| ΔnuoIJΔ nqrΔ ndh | expresses no known NADH dehydrogenase |
| Δanr |  |
|  |  |
| Chromosomal Complementation Strains |  |
| Δ *nuo IJ*::pUC18-miniTn7-P_ntp2_-nuoIJ | GmR, made in both PAO1 and PA14 |
| Δ*nuoIJ*::pUC18-miniTn7-P_ntp2_-ndh | GmR, made in both PAO1 and PA14 |
| ΔroxSR::pUC18-miniTn7-roxSR | with endogenous promoter, GmR, made in PAO1 |
|  |  |
|  | |
|  |  |
|  |  |
|  |  |
|  |  |
|  |  |
